# Supplementary material for: From sequence to enzyme mechanism using multi-label machine learning
Source: BMC Bioinformatics. 2014 May 19;15:150. doi: 10.1186/1471-2105-15-150 (PMC4229970; doi:10.1186/1471-2105-15-150)
Supplement: Additional file 2 — Java code of ml2db. Additional file ml2db_code.tar.gz contains the Java source code to run the multi-label machine learning experiments and save the results to database. The code’s Javadoc is included. [file 1471-2105-15-150-S2.zip › additional file 2/ml2db/utils/doc/uk/ac/standrews/utils/main/webutils/WebUtils.html]

WebUtils


JavaScript is disabled on your browser.


- Overview
- Package
- Class
- Use
- Tree
- Deprecated
- Index
- Help

- Prev Class
- Next Class

- Frames
- No Frames

- All Classes

- Summary:
- Nested |
- Field |
- Constr |
- Method

- Detail:
- Field |
- Constr |
- Method


uk.ac.standrews.utils.main.webutils

## Class WebUtils

- java.lang.Object
- - uk.ac.standrews.utils.main.webutils.WebUtils

- ---

    

  ```
  public class WebUtils
  extends java.lang.Object
  ```

  Web utilities. For reading from URL etc.

  Version:
  :   12 Dec 2007

  Author:
  :   Luna De Ferrari luna.deferrari-at-ed.ac.uk

- - ### Field Summary

    Fields

    | Modifier and Type | Field and Description |
    | --- | --- |
    | `static boolean` | `m_debug` |
  - ### Constructor Summary

    Constructors

    Constructor and Description

    `WebUtils()`
  - ### Method Summary

    Methods

    | Modifier and Type | Method and Description |
    | --- | --- |
    | `static java.io.BufferedReader` | `getReaderFromInputStream(java.io.InputStream stream)` Creates a buffered reader from an input stream |
    | `static java.io.InputStream` | `getStreamFromFileUri(java.lang.String url)` |
    | `static java.util.zip.GZIPInputStream` | `getStreamFromGzipUrl(java.lang.String gzipUrl)` |
    | `static java.io.InputStream` | `getStreamFromURL(java.lang.String url)` Creates an input stream from a URL (gets all the HTML or XML contained in the page) |
    | `static java.lang.String` | `getStringFromBufferedReader(java.io.BufferedReader reader)` Get a string from a buffered reader |
    | `static java.lang.String` | `getStringFromUrl(java.lang.String url, boolean gzip)` Get a string from a URL |
    | `static java.lang.String` | `md5Hash(java.lang.String s)` Create the md5 hash of a text From http://snippets.dzone.com/posts/show/3686 |

    - ### Methods inherited from class java.lang.Object

      `equals, getClass, hashCode, notify, notifyAll, toString, wait, wait, wait`

- - ### Field Detail


    - #### m\_debug

      ```
      public static boolean m_debug
      ```
  - ### Constructor Detail


    - #### WebUtils

      ```
      public WebUtils()
      ```
  - ### Method Detail


    - #### getReaderFromInputStream

      ```
      public static java.io.BufferedReader getReaderFromInputStream(java.io.InputStream stream)
      ```

      Creates a buffered reader from an input stream

      Parameters:
      :   `url` - a URL

      Returns:
      :   reader

      Throws:
      :   `java.io.IOException`


    - #### getStreamFromFileUri

      ```
      public static java.io.InputStream getStreamFromFileUri(java.lang.String url)
      ```


    - #### getStreamFromGzipUrl

      ```
      public static java.util.zip.GZIPInputStream getStreamFromGzipUrl(java.lang.String gzipUrl)
      ```


    - #### getStreamFromURL

      ```
      public static java.io.InputStream getStreamFromURL(java.lang.String url)
                                                  throws java.io.IOException
      ```

      Creates an input stream from a URL (gets all the HTML or XML contained in
      the page)

      Parameters:
      :   `url` - a URL or file URI

      Returns:
      :   an input stream object

      Throws:
      :   `java.io.IOException`


    - #### getStringFromBufferedReader

      ```
      public static java.lang.String getStringFromBufferedReader(java.io.BufferedReader reader)
      ```

      Get a string from a buffered reader

      Parameters:
      :   `reader` - a reader object

      Returns:


      Throws:
      :   `java.io.IOException`


    - #### getStringFromUrl

      ```
      public static java.lang.String getStringFromUrl(java.lang.String url,
                                      boolean gzip)
      ```

      Get a string from a URL

      Parameters:
      :   `url` -
      :   `gzip` - whether the document in the url has to be gunzipped

      Returns:


    - #### md5Hash

      ```
      public static java.lang.String md5Hash(java.lang.String s)
      ```

      Create the md5 hash of a text From
      http://snippets.dzone.com/posts/show/3686

      Parameters:
      :   `s` - the text to hash

      Returns:
      :   the md5 code

      Throws:
      :   `java.security.NoSuchAlgorithmException`


- Overview
- Package
- Class
- Use
- Tree
- Deprecated
- Index
- Help

- Prev Class
- Next Class

- Frames
- No Frames

- All Classes

- Summary:
- Nested |
- Field |
- Constr |
- Method

- Detail:
- Field |
- Constr |
- Method
